# In depth analysis of risk factors for coeliac disease amongst children under 18 years Old in the Gaza strip. A cross sectional study

**DOI:** 10.1186/1475-2891-11-97

**Published:** 2012-11-20

**Authors:** Mohammad B Al-Raee, Mazen A El-Sakka, Adnan A Al-Wahaidi

**Affiliations:** 1 El Dorra Pediatrics Hospital, Yafast, the Gaza strip, Gaza city, Palestine; 2 Collage of Pharmacology, Al-Azhar University, Al-Thalathiney St, the Gaza strip, Gaza city, Palestine; 3 Ard El-Insan Palestinian Benevolent Association, Al-Nussir St, the Gaza strip, Gaza city, Palestine

**Keywords:** Ard El-Insan: AEI, Body Mass Index: BMI, Coeliac Diseases: CD, Enteropathy, Endomyseal antibodies: EMA, Gluten, Tissue Transglutaminase: TtG

## Abstract

Coeliac disease is an important clinical disorder affecting the human gastrointestinal tract leading to multiple signs and symptoms in different body organs. This disease was the subject of a cross sectional descriptive-analytic study conducted in the Gaza Strip during 2010. Objectives were oriented to identify and verify several variables and attributes affecting the prognosis of coeliac disease in the patients. Ninety five children out of 113 patients were arranged into two groups according to age from 2 to 11 years and from 12 to 18 years old. Results showed the poor interest of health professionals regarding coeliac disease in the Gaza Strip. The mean age of study population was 5.47 years for males and 8.93 years for females. The lifestyle of coeliac patients was directly proportional with better nutritional indictors. Poor recognition of the emblem illustrating gluten in foods implicates effective health awareness or promotion. The more knowledgeable patients or mothers (P = 0.036) were the more compliant. The compliance to giving gluten free foods outside home was statistically significant (P = 0.037). Similarly, cautious approach when buying foods or detergents (P = 0.011). According to BMI 74.4%, 23.4% and 3.2% of all patients were normal, underweight and overweight respectively. Albumin blood level was normal in 32.6% and low in 67.4%. Meanwhile, blood calcium level was normal in 76.8%, low in 21.1% and high in 2.1% of all patients. **Conclusion**: The study showed that recreation and social activities for coeliac patients are substantially missing in the Gaza Strip. Moreover, the study proved that AEI is a reliable centre for care of coeliac disease patients and conducting relevant studies. Recommendation: There is a need for thorough and continuous community and institutional mobilization regarding coeliac disease in the Gaza Strip and in Palestine.

## Introduction

Coeliac disease is an organic disease, which affects the small bowel mucosa causing disturbance of absorptive functional capacity of small intestine. The disease appears in predisposed individuals as a result of dietary exposure to gluten. The classical nature of coeliac disease is characterized by mucosa inflammation, villous atrophy, and crypt hyperplasia. Clinical and histological finding show an improvement coupled with withdrawal of gluten from the diet [1].

Coeliac disease is also known as Coeliacsprue, gluten-sensitive enteropathy or non tropicalsprue [2]. The disease develops as a response to the dietary ingestion of glutens (prolamins and glutenins) of wheat and similar proteins in barley, rye and oats resulting in activation of both cell-mediated (T-cell) and humoral (B-cell) immune response in genetically susceptible persons [3].

Genetic predisposition is suggested by a high concordance amongst monozygotic twins approaching 70%, and in association with certain type II Human Leukocyte Antigens (HLA). Between 75% to 95% of HLA DQ2 is found in coeliac disease patients, whilst, most of the remaining patients have HLA DQ that are located in number six chromosome [4].

Environmental factors may also play a role in expression of coeliac disease whereas; some other factors may play a role in precipitating the symptoms. These include viral infection, high dose of gluten or early gluten challenge and pregnancy [5].

Coeliac patients are also specifically characterized by the presence of auto-antibodies to a connective tissue element surrounding smooth muscle called endomysium, which is highly specific for coeliac disease and known as endomyseal antibodies. Furthermore, an enzyme called tissue transglutaminase (tTG) may play a role in the pathogenesis of coeliac disease by modifying gluten peptides [6].

Coeliac disease appears actually in different clinical pictures and presentations.

Classical coeliac disease is gluten induced villous atrophy and presents in classical picture of intestinal malabsorption. Atypical coeliac disease might present in other different pictures such as decreased calcium levels, iron deficiency, osteoporosis, short stature, miscarriage and infertility. Silent coeliac disease might be discovered by serological screening or endoscope done to investigate other causes that are not related to the disease itself. Another form of the disease is known as latent coeliac disease characterized with delayed appearance of signs and symptoms of the disease, and it shows improvement on a gluten-free diet and appearance of normal mucosal histology [7].

Prevalence of coeliac disease is difficult to be estimated because of the different forms of clinical picture and variety in presentation of the disease itself, especially in patients who have mild form of the disease or those who have no apparent signs or symptoms. The highest prevalence of disease was estimated among Celtic populations and It was 1:300 to1:80 [8]. A high prevalence of coeliac disease was estimated in European populations and the incidence was slightly high amongst girls (57%). Sub clinical or silent coeliac disease was most abundant [9]. In the general population of developed societies it is estimated that prevalence of coeliac disease ranged mostly from 0.5% to 1%. Whilst, in the Sahara population who are African descendants of Arab-Berber and live in great African Desert “Sahara” the highest records of coeliac disease were reported and it appeared to be 5.6%, which was almost five to ten times more frequent than that in Europe [10].

In the Gaza strip, the already diagnosed cases of coeliac disease have been estimated at 237 patients i.e. 0 02% of the total population in the Gaza strip [11].

The diagnosis of coeliac disease is classically based on clinical suspicion or atypical presentations. Compatible further many serologic markers and duodenal biopsy is the most reliable diagnostic procedure in coeliac disease.

The major complications of coeliac disease include intestinal T-cell lymphomas and extra intestinal malignancies like Non Hodgkin lymphoma and esophageal cancer. Poor dietary compliance on the long run is associated with long term complications such as chronic malnutrition, decreased blood calcium level, neurological complications, miscarriage, congenital malformations, and low birth weight of babies. Those complications are responsible for the bad prognosis of patients. It is actually improved by taking gluten free diet [12].

Changing lifelong eating habits and adapting new gluten free lifestyle can be a big challenge for most people with coeliac disease because gluten free diet is expensive and does not taste as good as regular foods. Hectic nutritional lifestyles may resXult in insidious intake of gluten which might be included or hidden in foods or medications that possibly contain wheat or other grains. Good dietary compliance decreases the risk for future complications and improves life of coeliac patients [13].

## Methods and subject

### Study design and population

A cross sectional study was conducted during June 2009 on 113 children 2-18 years old diagnosed with coeliac disease in Ard El-Insan clinic in the Gaza Strip of Palestine, with a response rate of 85%. Parents, or first degree relatives, were interviewed when children 2-11 years old were unfit for responding to questionnaire [14].

Ethical approval and a consent form were obtained from authorized national personnel and institutions. Whilst, inclusion and exclusion criteria involved children age, diagnosis, residency, availability of age relevant caregiver, mental fitness and cooperativeness.

### Study instruments of data collection

Document revision and interview questionnaires with the patient, caring parent or first degree relatives were performed to obtain information about knowledge, attitude, compliance and socio-demographic factors. Measurements of weight and height were recorded for each interviewed patient using the device Seca 700, followed with collection of 5 ml. venous blood samples in order to test blood calcium and albumin levels. Body mass index (BMI) was calculated and recorded considering the reference values of normality, underweight as <5% tiles and overweight as >85% tiles [15]. Body weight and height were also calculated considering the child's age and cooperative attitude. Figures were recorded in kilograms to the nearest 100 grams and in centimeters to the nearest Figures are instantly recorded and double checked then the scale is recalibrated to the baseline (zero) reading. If the child is rather irritable the tarred (present in AEI clinic) weighing procedure was used instead [16]. Children were measured for weight and height with minimal clothes, without shoes, thick socks, head covers or hair ornaments and considering the upright standing position [16].

Blood samples were tested for total calcium and serum albumin via “Chem-Well” blood analyzer machine.

### Data analysis and processing

Data collected and admitted was analyzed using the SPSS version 13 statistical package of health information scientific analysis in order to obtain precisely accurate results. Statistical significance had been used via p value less than 0.05. Pilot study of 20 patients was conducted before starting the real data collection in order to induce necessary amendments.

### Constraints of the study

The study went on smoothly despite all the inevitable constraints that had been encountering the performance in the already vulnerable Gaza Strip.

## Results and discussion

A- Socio-Demographic Factors Table 1

**Table 1:** Socio-demographic factors and blood calcium level in all patients

**Socio-demographic factors**	**Blood calcium level**		***p-value***
	**Low range < 8.8mg/dl n=22 (%)**	**Normal range ≥ 8.8mg/dl n=73 (%)**	
**Sex**			
Male	12 (35.3)	22 (64.7)	*0.036
Female	10 (16.4)	51 (83.6)	
**Mother education**			
Primary	5 (29.4)	12 (70.6)	0.306
Preparatory	6 (31.6)	13 (68.4)	
Secondary	11 (21.6)	40 (78.4)	
Over secondary	0 (0.0)	8 (100.0)	
Over secondary	4 (13.3)	26 (86.7)	
**Monthly income**			
<400$	21 (33.3)	42 (66.7)	*0.004
400-700$	1 (3.2)	30 (96.8)	
>700$	0 (0.0)	1 (100.0)	
**House status**			
Owned	17 (19.8)	69 (80.2)	*0.015
Rented	5 (55.6)	4 (44.4)	

Mother education is not statistically associated with blood calcium level, whilst differences in blood calcium level were statistically significant in the two sexes (p = 0.036). Monthly income and house statues constituted [17] an identifier of the significant differences of blood calcium level (p = 0.004 and 0.015) as attribute to the effect of low socioeconomic status and a possibly the unfavourable rented home environment on blood calcium level [18], possibly poor nutrition, lack of exposure to sun, missing the healthy environment and sanitation (Table 2).

**Table 2:** Distribution of study sample by knowledge in two age groups

**Life quality and cure of coeliac disease**			
**Indicators**	**Age group (%)**		**p-value**
	**2-11Yrs n = 66 (%)**	**>11-18Yrs n = 29 (%)**	
**Believe coeliac patients have a normal life**			
No	23 (34.8)	8 (27.6)	0.785
Don’t know	4 (6.1)	2 (6. 9)	
Yes	39 (59.1)	19 (65.5)	
**Coeliac disease can be cured completely**			
No	14 (21.2)	11 (37.9)	*0.011
Don’t know	14 (21.2)	11 (37.9)	
Yes	38 (57.6)	7 (24.1)	

Knowledge discrepancy exists between the older and younger ones whose answers were made by their mothers or care takers. This reflects an optimistic thinking appearing clearly regarding knowing life normality (p = 0.785) and curability of coeliac disease (p = 0,011) in the youngest group. This might be an attribute to an individual negative feeling rather than being a real knowledge. If their knowledge is getting better, they’ll present with more positive answers about life quality expectations and more realistic answers regarding curability.

**Figure 1:**
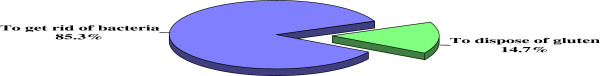
Knowledge about importance of hand washing after dealing with gluten substances.

An important relevant notion regarding the patient’s general health and consequences of coeliac disease appeared in the study when parents of compliant coeliac patients were less worried about their children’s health in general. But, they were very much concerned about the possible adverse effects of coeliac disease that might affect the future life of their children [19] (Figure 1).

When asked about hand washing, information on gluten contamination was little. 14.7% practiced hand washing to avoid possible gluten contamination whilst, 85.3% claimed disinfection and cleansing (Table 3).

**Table 3:** Embarrassment being a coeliac patient and consequences

**Indicators**	**Age group (%)**		**p-value**
	**2-11Yrs n = 66 (%)**	**>11-18Yrs n = 29 (%)**	
**Embarrassment, if others know about patient’s or son’s sickness with CD**			
No	46 (69.7)	15 (51.7)	0.092
Yes	20 (30.3)	14 (48.3)	
**Whether embarrassment in front of others impair compliance to gluten free food**			
Yes	13 (65.0)	6 (46.2)	0.284
No	7 (35.0)	7 (53.8)	

No tatistical significance exists when comparing the attitude of patients or caregivers towards embarrassment arising from being discovered as coeliac patients or the effect of this on compliance to gluten free foods in front of others. This is possibly an attribute to psychological and cultural components. Results were similar in one component to the study conducted in Canada 2003 showing that, 53% of coeliac patients were embarrassed enough to find difficulty in bringing and eating gluten free foods in the public [20] (Table 4).

**Table 4:** Behaviors denoting compliance to gluten free foods

**Compliance indicators**	**Age group (%)**		**p-value**
	**2-11Yrs n = 66 (%)**	**>11-18Yrs n = 29 (%)**	
**Gluten-free foods taken outside home**			
Yes	59 (89.4)	21 (72.4)	*0.037
No	7 (10.6)	8 (27.6)	
**Caution regarding gluten when buying food and detergent**			
Yes	53 (80.3)	24 (82.8)	*0.011
No	12 (18.2)	1 (3.4)	
Don’t know	1 (1.5)	4 (13.8)	
Give special meal (Gluten free)	63 (95.5)	26 (89.7)	

Statistical significant results are observed (p = 0.037 and 0.001 respectively) regarding compliance to taking gluten free foods outside home and caution when buying foods and detergents to avoid gluten contamination. More care was observed amongst the youngest group denoting mother's or care giver's protection offered and cautious actions followed. This result applies with statistical figures of a Canadian study on coeliac disease in which, 95% of coeliac children either take with them or be given the gluten free foods by their families in order to be eaten outside homes [21] (Table 5).

**Table 5:** Body mass index distributed in study sample

**Indicators**	**Number**	**%**
**Body mass index (BMI** percentile**)**		
Normal BMI (5th -85th percentile)	69	72.6
Underweight (< 5th percentile)	22	23.2
Overweight (≥ 85th percentile)	4	4.2

### Anthropometric and biochemical indicators

Discrepancy in BMI amongst patients emphasizes the notion that controlled coeliac disease would be possibly a reason for improvement of underweight condition (23.2%) and even normality of BMI (72.6%). Increased BMI up to obesity might be explained by other factors not necessarily relating to coeliac disease. Results in other studies showed that obese or overweight coeliac patient’s actually loose weight and improve in terms of BMI after initiation of treatment based on gluten free diet [22] (Table 6).

**Table 6:** Calcium and albumin blood level distributed in study sample

**Indicators**	**Number**	**%**
**Albumin level in the blood**		
Normal (3.9-5.3g/dl)	31	32.6
Low Albumin (<3.9g/dl**)**	64	67.4
**Calcium level in the blood**		
Normal Calcium (8.8-10.8mg/dl)	71	74.7
Low Calcium l (<8.88mg/dl)	22	23.2
Higher Calcium (**>**10.8mg/dl	2	2.1

**Figure 2:**
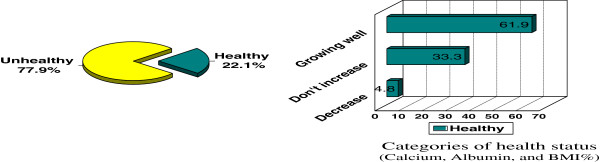
Patient's health status described as healthy by children or their mothers against calcium, albumin and BMI in the corresponding health scale.

The apparently decreased albumin level in 67.4% of coeliac patients coupled with 23.2% [18] of low calcium level in the same group give a clue on the pathogenesis of coeliac disease and its direct relation with some abnormal values of biological indicators and elements Figure 2).

**Figure 3:**
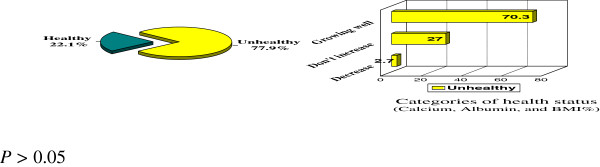
**Patient's health status described as unhealthy by children or their mothers against calcium, albumin and BMI in the corresponding health scale.***P >* 0.05.

Amongst the 22.1% of healthy coeliac patients 61.9% were truly growing well whilst 33.3% of the same group claimed an adverse opinion being not increasing in weight which is not true (Figure 3).

Controversial opinions attributable to personal attitudes and knowledge regarding health status of coeliac diseased individuals appeared when 70.3% were proved to be unhealthy despite saying that they were healthy and growing well. Such a contradiction between self and technically (clinically evidence based) evaluated ones emphasizes the fact that technical reliability is the best trend of evaluation and not to rely on patient’s or care giver’s opinion. Results were not statistically significant (P > 0.05).

## Conclusion

Coeliac disease exists in the Gaza Strip and requires further attention by health providers. Ard El-Insan is the only organization which supports coeliac patients who are predominantly females. A questionable statistical significance exists between the blood calcium level of coeliac patients and several socio-demographic variables. To a lesser extent this could be similar to the relationship between BMI and mother education, whilst albumin level has been proved to be a sensitive indicator of noticeably low values in coeliac patients that requires higher attention.

BMI was normalized when using gluten free diet in obese or overweight patients.

High percentage of patients and mothers has contradicting ideas and notions regarding the life quality of patients with coeliac disease.

Most of patients lack the knowledge about emblem of coeliac disease. As regards knowing the gluten containing foods there had been more knowledge amongst mothers than the patients themselves especially in the older groups. Contamination of foods with gluten from different sources or food procedures inside and outside homes was poorly recognized by the patients and their care givers.

Unified knowledge pattern regarding coeliac disease is a matter of concern in many aspects relating to morbidity and mortality components as inferred from patient’s opinions regarding life quality of coeliac patients.

Feeling stigmatized by patients or their mothers was translated into embarrassment when giving their children gluten free diet outdoors or in doors.

High level of compliance towards gluten free food and medicines or detergents.

BMI, Albumin and calcium blood levels were important indicators in determining the progress of coeliac disease as linked with other etiological correlations including socio demographic elements.

Other clinical signs and symptoms of coeliac disease are important factors to judge upon the reliability of patient’s answer.

## Recommendations

• The following recommendations were considered by the researches as an outcome of this study.

• More care in coeliac disease is recommended to be practiced in the primary, secondary and tertiary health care services by all health providing sectors.

• Further investigations, researches and studies are necessary to facilitate building up policies for early case detection and management of coeliac disease in the Gaza Strip.

• Health staff in all sectors should be encouraged, prepared and promoted in order to contribute effectively to the process of management of coeliac disease.

• Encouragement of social activities and community participation of coeliac patients and their families are requested.

• Development of the program in AEI and other healthy organization to support patients psychologically.

• More awareness at community and institutional levels about coeliac disease.

• MOH and other health sectors should be ready to offer diagnostic facilities.

• More health awareness and counseling on recommended gluten free diet.

• More investigation of albumin blood level in coeliac patients and follow up are required.

• Screening for at risk approach individual.

## Competing interests

The authors declare that they have no competing interests.

## Authors' contribution

M.A. Selected the topic and assigned the target group, place and type of the study as a cross sectional descriptive, setting the conclusion and references. M.S. Shared in structuring the research, revising the document and finalization of the study. A.W. Developed methodology, helped elaborating results, discussion and formulation the recommendations. All authors read and approved the final manuscript.
